# Central Retinal Vein Occlusion with Therapeutic Level of Anticoagulation

**DOI:** 10.1155/2009/827982

**Published:** 2009-06-18

**Authors:** Sarwat Salim, Wai-Ching Lam, Wahid Hanna

**Affiliations:** ^1^Hamilton Eye Institute, University of Tennessee Health Science Center, 930 Madison Avenue, Memphis, TN 38163, USA; ^2^Department of Ophthalmology, University of Toronto, Toronto, ON, Canada M5T 2S8; ^3^Department of Hematology, University of Tennessee, Knoxville, TN 37920, USA

## Abstract

*Purpose*. To describe a patient with two episodes of deep venous thrombosis and factor V Leiden mutation who presented with central retinal vein occlusion (CRVO) despite prophylactic use of warfarin sodium (Coumadin). *Methods*. A case report of a 44-year-old woman with a history of recurrent deep venous thrombosis and Factor V Leiden mutation was placed on lifelong prophylactic therapy with warfarin. The patient presented with CRVO in the left eye despite therapeutic levels of warfarin. *Results*. Extensive systemic evaluation disclosed high titers for antinuclear antibody (ANA). *Conclusion*. Systemic anticoagulation with warfarin may not preclude further thrombotic episodes. In younger patients presenting with retinal vein occlusion and pre-existing multiple thrombophilic risk factors, a multidisciplinary approach is recommended to explore other therapeutic options to avoid further thromboembolic complications.

## 1. Introduction

Vascular occlusive disease of the retina has various etiologies. In the older population, systemic vascular diseases such as diabetes and hypertension are common. In younger patients, inherited coagulation abnormalities are usually responsible [1]. We report a case of a young woman who presented with CRVO due to a hypercoagulable state secondary to factor V Leiden mutation, despite systemic anticoagulation therapy.

## 2. Case Description

A 44-year-old woman with a history of recurrent deep venous thrombosis was started on lifelong warfarin therapy after being diagnosed with factor V Leiden mutation. Three years later, she presented with acute visual loss in the left eye. Examination revealed visual acuity of 20/30 OD and 5/200 OS. Slit lamp examination was unremarkable. Fundus examination of the right eye was normal and in the left eye, retinal hemorrhages and dilated retinal veins were present in all four quadrants along with macular edema.

Systemic evaluation disclosed normal serum electrolytes and blood cell count. The INR was reported to be in therapeutic range, 2.48. Anticardiolipin antibodies and lupus anticoagulants were negative but high titers of ANA were documented (1 : 320 with speckled pattern). The rheumatology service decided to monitor the patient closely for development of systemic lupus erythematosis (SLE) given high titers of ANA.

## 3. Comment

Factor V Leiden mutation is known to be the most common cause of inherited thrombophilia and has been shown to be a causative factor in CRVO [1]. This point mutation involving an amino acid substitution, where arginine is converted to glutamine at position 506, results in resistance to activated protein C, a protease that plays a vital role in physiological anticoagulation by inactivating the coagulant factors V and VIII. 

Thromboembolic complications are frequently observed in patients with SLE, and the risk of thrombosis is further potentiated in the presence of Factor V Leiden mutation [2]. Patients with SLE, even in the absence of antiphospholipid antibodies, carry a six fold risk of venous thromboembolism when compared to the general population.

Browning and Fraser [3] reported occurrence of CRVO in eyes despite the use of systemic warfarin; however, the majority of their patients had low INRs relative to the indication for anticoagulation (INR < 2). In contrast, Mruthyunjaya et al. [4] reported occurrence of CRVO with variable INR values, concluding that therapeutic range of INR is important to prevent secondary systemic thrombotic and embolic disease. Our patient developed CRVO despite the recommended therapeutic level of warfarin. 

Management of young patients with multiple thrombophilic risk factors continues to be a tremendous challenge for clinicians. Although the use of concomitant anticoagulation and antiplatelet therapy (aspirin, clopidogrel) remains controversial, some have suggested a higher INR with anticoagulation and/or addition of antiplatelet therapy to avoid further thromboembolic morbidity and mortality. The use of various classes of medications with different mechanism of action may provide some benefit; however, additional studies are needed to substantiate these recommendations [5]. One needs to be mindful of increased risk of bleeding, and therefore, the need for a combination therapy must be individualized using a multidisciplinary approach with various subspecialists, including hematologists.
